# The functional expression and characterisation of a cysteine peptidase from the invasive stage of the neuropathogenic schistosome *Trichobilharzia regenti*^☆^

**DOI:** 10.1016/j.ijpara.2008.06.010

**Published:** 2009-01-15

**Authors:** Kateřina Dolečková, Martin Kašný, Libor Mikeš, Jared Cartwright, Petr Jedelský, Eric L. Schneider, Jan Dvořák, Adrian P. Mountford, Charles S. Craik, Petr Horák

**Affiliations:** a Department of Parasitology, Faculty of Science, Charles University in Prague, Viničná 7, 12844 Prague 2, Czech Republic; b Department of Biology (Area 5), The University of York, YO10 5YW York, UK; c Laboratory of Mass Spectrometry, Faculty of Science, Charles University in Prague, Viničná 7, 12844 Prague 2, Czech Republic; d Department of Pharmaceutical Chemistry, University of California San Francisco, CA 94158, USA; e Sandler Center for Basic Research in Parasitic Diseases, University of California San Francisco, CA 94158, USA

**Keywords:** Trematoda, Cathepsin B, Protease, Penetration gland, Skin, Histolysis, *Schistosoma*, Cercaria

## Abstract

A transcriptional product of a gene encoding cathepsin B-like peptidase in the bird schistosome *Trichobilharzia regenti* was identified and cloned. The enzyme was named TrCB2 due to its 77% sequence similarity to cathepsin B2 from the important human parasite *Schistosoma mansoni*. The zymogen was expressed in the methylotropic yeast *Pichia pastoris*; procathepsin B2 underwent self-processing in yeast media. The peptidolytic activity of the recombinant enzyme was characterised using synthetic fluorogenic peptide substrates at optimal pH 6.0. Functional studies using different specific inhibitors proved the typical cathepsin B-like nature of the enzyme. The S_2_ subsite specificity profile of recombinant TrCB2 was obtained. Using monospecific antibodies against the recombinant enzyme, the presence of cathepsin B2 was confirmed in extracts from cercariae (infective stage) and schistosomula (early post-cercarial stage) of *T. regenti* on Western blots. Also, cross-reactivity was observed between *T. regenti* and *S. mansoni* cathepsins B2 in extracts of cercariae, schistosomula or adults. In *T*. *regenti*, the antisera localised the enzyme to post-acetabular penetration glands of cercariae implying an important role in the penetration of host skin. The ability of recombinant TrCB2 to degrade skin, serum and nervous tissue proteins was evident. Elastinolytic activity suggests that the enzyme might functionally substitute the histolytic role of the serine class elastase known from *S*. *mansoni* and *Schistosoma haematobium* but not found in *Schistosoma japonicum* or in bird schistosomes.

## 1 Introduction

*Trichobilharzia regenti* is a nasal avian schistosome related to important human parasites of the genus *Schistosoma*. Like those, bird schistosomatids have complex two-host life cycles. Cercariae shed into the water environment from an intermediate snail host must find and penetrate the skin of a final host. Ultimately, cercariae transform into schistosomula, which in the case of the uniquely neurotropic *T*. *regenti* migrate through the peripheral nerves and CNS causing severe pathologies in birds and even in mammals (Kolářová et al., 2001). The parasites mature, mate and lay eggs only in the nasal cavity of anatid birds (Horák et al., 1999; Hrádková and Horák, 2002). Occasionally, cercariae of bird schistosomes can also penetrate into human skin during bathing and cause an allergic reaction manifested as cercarial dermatitis (swimmer’s itch) (Horák and Kolářová, 2001; Kouřilová et al., 2004; Horák et al., 2002), which is considered an emerging disease in many parts of the world.

Throughout their life cycle, schistosomes employ peptidases for many indispensable tasks, including host-tissue penetration (Yoshino et al., 1993; Salter et al., 2000, 2002; McKerrow and Salter, 2002), metabolism of host macromolecules for parasite nutrition (Caffrey et al., 2004; Delcroix et al., 2006) and evasion or manipulation of host immune responses (Marikovsky et al., 1990; Cocude et al., 1999). Recently, our interest has focused upon cysteine peptidases of parasitic organisms because of their critical importance to parasite survival, making them valuable targets for rational design of new efficient anti-parasite drugs (Rosenthal et al., 2002; Renslo and McKerrow, 2006; Abdulla et al., 2007; Reis et al., 2007). In medically important *Schistosoma mansoni*, cathepsin B1, the predominant cysteine peptidase among several gut-associated digestive enzymes (Caffrey et al., 2004), is considered an attractive target for anti-parasite chemotherapy (Abdulla et al., 2007). In addition, this enzyme is able to elicit an antibody response in hosts and its immunogenicity has been used as one of the main serodiagnostic markers for schistosomiasis (Li et al., 1996).

Most studies on human schistosomes have focused on blood-dwelling developmental stages (schistosomula and adults), but less is known about cercarial cysteine peptidases. In cercariae of *S. mansoni*, cathepsins B and L have been reported to be present in post-acetabular penetration glands and supposed to be involved in disruption of the outer keratinised layer of the skin (Dalton et al., 1996, 1997). Also Kašný et al. (2007) demonstrated cysteine peptidase activity of cathepsin B-type in secretions of cercarial penetration glands. However, Skelly and Shoemaker (2001) localised cathepsin B1 to the protonephridial system and caeca of *S*. *mansoni* cercariae.

Until recently, a single Clan PA chymotrypsin-like serine peptidase with elastinolytic activity has been considered as the main penetration tool of schistosome cercariae (Salter et al., 2000, 2002). However, this was found not to be the case for *Schistosoma japonicum* (Dvořák et al., 2008). Therefore, differences in the enzymatic equipment used for penetration between different species of schistosomes require more detailed examination (Pleass et al., 2008). Our previous studies showed that no orthologue of *S*. *mansoni* elastase is present in *Trichobilharzia* and instead cysteine peptidase activity dominates in the cercariae. Two cysteine peptidases of 31 kDa and 33 kDa have been identified in the excretory/secretory (E/S) products of *Trichobilharzia szidati* and *T. regenti* cercariae, respectively (Mikeš et al., 2005). In the latter species, Kašný et al. (2007) showed that the major peptidase activities are of cysteine peptidase origin – cathepsin B and, to a certain degree, cathepsin L. The cathepsin B-like activity was present in praziquantel-induced secretions of penetration glands. In a recent study, Dolečková et al. (2007) retrieved a full-length cDNA sequence coding for cathepsin B1 from a mixed cDNA library based on intramolluscan stages (sporocysts and developing cercariae) of *T*. *regenti*; this was 100% homologous to the sequence of the TrCB1.1 isoform expressed in the gut of schistosomula (Dvořák et al., 2005).

In the present study, we have identified and cloned a cDNA sequence encoding a cathepsin B-like peptidase from cercariae of *T. regenti*. It was orthologous to *S. mansoni* and *S*. *japonicum* cathepsin B2 genes (GenBank Accession Nos. AJ312106 and AY226984), showing almost 80% sequence similarity and is therefore termed TrCB2. We performed overexpression in *Pichia pastoris* and characterised the physico-chemical properties and peptide substrate preferences of the recombinant enzyme. By means of immunohistochemistry, we show the enzyme to be present in cercarial post-acetabular penetration glands and, finally, we demonstrate the ability of TrCB2 to cleave relevant tissue proteins.

## 2 Materials and methods

### 2.1 Parasites

*Trichobilharzia regenti* has been routinely maintained under laboratory conditions (Horák et al., 1998). Infected snails were dissected and the entire digestive gland, containing both snail tissues and parasitic larval stages (sporocysts with developing cercariae) excised and used as an initial source of total RNA. Fresh fully developed cercariae emerging from snails were collected (Kašný et al., 2007), while post-cercarial stages of the parasite (schistosomula) were obtained from infected ducks 8 days p.i. as described by Dvořák et al. (2005). Live *S*. *mansoni* adults were obtained from the Institute for Postgradual Medical Education, Prague, whereas lyophilised *S*. *mansoni* cercariae were from the School of Biological Sciences, University of Wales, Bangor, UK. Animal treatment was in concordance with the legislation of the Czech Republic and the European Union.

### 2.2 Production, cloning and sequencing of TrCB2 cDNA

Using TRIzol (Invitrogen, USA), total RNA was isolated from homogenate of 10 infected snail hepatopancreases. Subsequently, mRNA was extracted using a MicroPoly(A)Purist mRNA Purification Kit (Ambion, USA). First-strand cDNA synthesis was carried out with 3 μg of mRNA using oligo-dT_18_ primer (Generi-Biotech s.r.o., Czech Republic) and Superscript II Reverse Transcriptase (Invitrogen, USA) following the manufacturer’s instructions. Obtained cDNA was used as a template for subsequent PCRs.

Two rounds of PCR amplifications were performed with degenerate oligonucleotide primers. The forward primer TrCBdegfrd 1(5′-TTYGGNGCNGTNGARGC-3′) was the same for both rounds of PCR, reverse primers TrCBdegrev 2(5′-TCNCCCCARTCRCTRTTCCA-3′) and TrCBdegrev 5(5′-TTNGCDATYAACCARTANGG-3′) were used in the first and second round of PCR, respectively. Amplified products of the expected size were gel purified using the MinElute Gel Extraction Kit (Qiagen), and cloned directly into the pCR^®^2.1-TOPO cloning vector (Invitrogen). Obtained construct was propagated in chemically competent *Escherichia coli* TOP10 cells, isolated using Qiaprep Purification Kit (Qiagen) and sequenced with the M13 forward and M13 reverse primers (DNA Sequencing Laboratory, Faculty of Science, Charles University in Prague). Full-length cDNA of cathepsin B was obtained by 5′ and 3′ rapid amplification of cDNA ends procedures by using the GeneRacer™ Kit (Invitrogen), and its nucleotide sequence was determined. To examine the presence of other TrCB2 isoforms, 10 PCR reactions were performed and the products subcloned into the pCR^®^2.1-TOPO cloning vector for propagation in *E. coli*. For each PCR, five clones were randomly selected for sequencing (50 clones in total).

BLASTp analysis (http://au.expasy.org/tools/blast/) and ClustalW alignment (http://au.expasy.org/tools/#align) of deduced amino acid sequences were conducted on ExPASy Proteomic Server of the Swiss Institute of Bioinformatics. The initial position of the TrCB2 pro-region was predicted with the SignalP software (Nielsen et al., 1997) at http://www.cbs.dtu.dk/services/SignalP.

### 2.3 Construction of yeast expression plasmid

The gene was amplified by PCR from the pCR^®^2.1-TOPO cloning vector (Invitrogen, USA) using the AccuPrime Pfx DNA polymerase Kit (Invitrogen). For amplification, sense primer KlonTrCB2fwd 5′-ATACTCGAG**AAAAGA**GAGGCTAATCGACACAAGTTTATG-3′, containing an XhoI restriction site (underlined) and Kex 2 peptidase cleavage site (in bold), and antisense primer KlonTrCB2rev 5′-ATGCGGCCGC**TTA**TTTAAGTTTCGGAATTCCAGC-3′, containing NotI restriction site (underlined) and a stop codon (in bold), were used. The 998-bp gene of procathepsin B2 (proTrCB2) was inserted into the XhoI/NotI double-digested expression vector pPICZα B (Invitrogen).

### 2.4 Expression and purification of recombinant TrCB2

The protocol of Dvořák et al. (2005) was adopted using wild type strain X33 of the methylotrophic yeast *P. pastoris* (Invitrogen) as a host strain. The cultures of selected transformed clones were grown for 2 days and centrifuged at 3,000*g* for 20 min; supernatants were clarified by filtration through a 0.22 μm filter. Complete buffer exchange with 50 mM sodium acetate pH 5 and approximately 250-fold concentration was accomplished using AmiconUltra 15 filters (Millipore; 10,000 MWCO). For purification of recombinant cathepsin B2, a Mono S cation-exchange column (Amersham Pharmacia) and fast protein liquid chromatography (FPLC; BioLogic, Bio-Rad) were used. The column was pre-equilibrated with 50 mM acetate buffer pH 5 at room temperature and proteins were eluted by use of a linear salt gradient (0–1 M NaCl). Obtained fractions were analysed by SDS–PAGE. The identity of the protein was confirmed by MALDI-TOF analysis of tryptic oligopeptides (Proteomics & Analytical Biochemistry, Biology Technology Facility, University of York, UK).

### 2.5 N-terminal amino acid sequence analysis

The purified recombinant TrCB2 was electrophoresed, blotted onto polyvinylidene fluoride (PVDF) membrane, stained by Coomassie brilliant blue and submitted for N-terminal Edman sequencing (Laboratory of Protein Sequencing, Institute of Organic Chemistry and Biochemistry, Academy of Sciences of the Czech Republic).

### 2.6 Deglycosylation of purified recombinant TrCB2

Mixture containing 10 μl of 5× reaction buffer (Native Protein Deglycosylation Kit; N-DEGLY, Sigma) and 200 μg (37.5 μl) of TrCB2 was incubated with 2 μl of Endoglycosidase F1 at 37 °C for 1 h. Deglycosylated TrCB2 was examined for a shift in molecular weight by SDS–PAGE analysis.

### 2.7 TrCB2 peptidase activity, inhibition assays and S_2_ subsite specificity

Typical assay conditions were as follows: TrCB2 (0.5 μg) in 100 μl of 50 mM citrate/100 mM phosphate buffer pH 6.0 (CPB) containing 5 mM DTT was mixed with 100 μl of 50 μM substrate in CPB. The pH optimum of TrCB2 activity was determined using buffers of pH in the range 3.0–8.0 and the cathepsin B-specific substrate Z-Arg-Arg-AMC. Peptidase activity was measured using fluorogenic aminomethylcoumarin-coupled (AMC) peptide substrates (Bachem): Z-Phe-Arg-AMC (to assay cathepsins B and L), Z-Arg-Arg-AMC (cathepsin B), Boc-Val-Leu-Lys-AMC (cathepsins B and L), Z-Gly-Pro-Arg-AMC (trypsin-like peptidases), Suc-Ala-Ala-Pro-Phe-AMC (chymotrypsin-like), Boc-Leu-Gly-Arg-AMC (trypsin-like). Assays were performed in black 96-well plates (Nunc, Denmark) using the Infinite M200 fluorometer (TECAN) at excitation and emission wavelengths of 355 and 460 nm, respectively. The release of AMC was measured within a period of 60 min.

Inhibition of the peptidolytic activity of both de- and glycosylated forms of TrCB2 was tested at pH optimum 6.0 with 100 μM Z-Arg-Arg-AMC, a substrate specific for cathepsin B and a spectrum of differentiating peptidase inhibitors at 50 μM concentrations: E-64 [*N*-*trans*-(epoxysuccinyl)-l-leucine 4-guanidinobutylamide], irreversible broad spectrum Clan CA cysteine peptidase inhibitor; CA-074 [*N*-(l-3-*trans*-propylcarbamoyloxirane-2-carbonyl)-Ile-Pro-OH], irreversible, selective, of cathepsin B (Towatari et al., 1991); Z-Phe-Ala-CHN_2_ [Z-Phe-Ala-diazomethylketone], irreversible, of cathepsins B and L (Dalton et al., 1997); Z-Phe-Phe-CHN_2_ [Z-Phe-Phe-diazomethylketone], irreversible, of cathepsins B and L (Nowak et al., 2004); Z-Phe-Tyr-CHN_2_, [Z-Phe-Tyr(tBu)-diazomethylketone], irreversible, of cathepsin L (Shaw et al., 1993); elastatinal [*N*-(Na-Carbonyl-Cpd-Gln-Ala-al)-Leu], irreversible, specific, of neutrophil and pancreatic elastases but not other serine peptidases such as trypsin or chymotrypsin (Bieth, 2004); DFP (diisopropylfluoro-phosphate), irreversible, of serine peptidases (Bharadwaj et al., 1996).

S_2_ subsite peptide substrate specificity of recombinant TrCB2 was examined using a positional scanning synthetic combinatorial library (PS-SCL) as described by Choe et al. (2006). Enzyme assays were carried out in CPB pH 6 containing 100 mM NaCl.

### 2.8 Production of antibodies against TrCB2, blotting and immunohistochemistry

#### 2.8.1 Antibodies

Antisera against glycosylated (anti-TrCB2) and deglycosylated (anti-degTrCB2) forms of recombinant TrCB2 were raised in 10-week-old BALB/c mice (ANLAB, Germany). The protein dissolved in 100 μl of sterile saline was mixed with an equal volume of TiterMax Gold^™^ adjuvant (Sigma) and emulsified by extensive sonication which also caused inactivation of protease activity. Two mice for each form of the enzyme were injected s.c. 3× at 10-day intervals (2 × 35 μg, second booster 25 μg of protein). Immune sera were collected 7 days after the last injection. Control sera were obtained from mice kept under the same conditions. For bleeding, mice were anesthetised by ketamine/xylazine. Animal treatment was in concordance with the legislation of the Czech Republic and the European Union.

#### 2.8.2 Western blotting

Cercariae and schistosomula of *T. regenti* and cercariae and adults of *S. mansoni* were homogenised by two cycles of sonication (7 W, 30 s each, Vibracell-72405, Bioblock Scientific) on ice in 10 mM PBS pH 7.2 and centrifuged at 13,000*g*, 4 °C for 10 min. The supernatants were used for SDS–PAGE. Cercarial extracts of *T. regenti* (200 μg) and *S. mansoni* (60 μg), schistosomular extract of *T. regenti* (100 μg), extract from adult *S. mansoni* (50 μg) or pure recombinant TrCB2 (10 μg) were separated by SDS–PAGE in 12% gels (Prep/2-D sample well comb) using the Mini PROTEAN^®^ 3 System (Bio-Rad) and electroblotted onto PVDF membrane. Blocking was performed by 5% non-fat dry milk in 20 mM Tris–HCl, 150 mM NaCl, pH 7.8, 0.05% Tween 20 (TBS-T) for 1 h. Membranes were incubated with mouse anti-TrCB2, anti-degTrCB2, rabbit anti-SmCB2, control mouse and/or rabbit non-immune sera (all 1:50 in 1% BSA/TBS-T) for 1 h, washed, incubated for 1 h with peroxidase-coupled goat anti-mouse or anti-rabbit IgG diluted 1:1,000 (Sigma), then finally washed and developed with Opti-4CN™ Substrate Kit (Bio-Rad).

#### 2.8.3 Ligand blotting

For peptidase-active site labelling, a biotinylated analogue of the Clan CA cysteine peptidase inhibitor E-64 (DCG-04) (Greenbaum et al., 2000) was used. Briefly, 15 μg of pure recombinant enzyme was incubated for 1 h at room temperature with 5 μM DCG-04 in 50 mM citrate/100 mM sodium phosphate pH 6.0 containing 5 mM DTT. Further, a previously described protocol was adopted (Mikeš and Man, 2003).

#### 2.8.4 Immunolocalisation of TrCB2

Cercariae of *T*. *regenti* were fixed, embedded and sectioned as described elsewhere (Mikeš et al., 2005). Blocking was performed in PBS containing 0.05% Tween 20 (PBS-T), 5% non-fat dry milk, laminarin (1 mg/ml) and a cocktail of peptidase inhibitors (Complete Mini Protease Inhibitor Cocktail tablets – Roche, Germany) in a wet chamber at 4 °C overnight. The slides were then incubated for 1.5 h with mouse sera (control or anti-TrCB2) diluted 1:50 in 2% BSA in PBS-T. After extensive washing, sections were incubated for 45 min with AlexaFluor-labelled anti-mouse IgG secondary antibodies (Molecular Probes) diluted 1:500. Sections were mounted into TBS-buffered glycerol (4:1) and examined using a confocal microscope equipped with the Acousto-Optical Beam Splitter system (Leica).

### 2.9 Degradation of natural substrates by recombinant TrCB2

The ability of TrCB2 to hydrolyse natural protein substrates was tested using collagen type IV (from human placenta), elastin (both insoluble and soluble from bovine neck ligament), fibrinogen type III (from human placenta, fraction I), keratin (from human epidermis), duck, turkey and bovine haemoglobins and myelin basic protein (from mouse brain).

The substrates (with the exception of elastin) at a final concentration of 0.2 mg/ml were incubated with recombinant TrCB2 (1 μg) in 50 μl of CPB containing 2 mM DTT at 37 °C overnight. Control reactions contained CPB instead of the enzyme. Aliquots (20 μl) of the resulting hydrolysates were mixed with non-reducing SDS–PAGE sample buffer, separated by electrophoresis in 15% SDS–PAGE and subsequently stained with Coomassie brilliant blue.

Degradation of insoluble elastin was tested by two different approaches. Suspensions containing 5.0 mg of insoluble elastin in 200 μl of CPB pH 6 were supplemented by 5 mM DTT. Reactions were started by addition of 1 μl (0.8 μg) of the enzyme and shaken in an Eppendorf Thermomixer Compact at 37 °C (1,200 rpm) to avoid sedimentation. After 2 h incubation, a modified procedure of Novinec et al. was applied (2007), employing fluorescamine for detection of N-termini of peptidase-generated peptides. Appropriate blanks were run to deduct the fluorescence developed by elastin and TrCB2 alone. The second experiment employed mass spectrometry identification of TrCB2-generated elastin peptides. An aliquot was taken from the samples after overnight incubation, concentrated on C18 ZipTips and applied directly on nano-reverse phase HPLC-MALDI-TOF-TOF (Laboratory of Mass Spectrometry, Faculty of Science, Charles University in Prague). Spectra were acquired in positive reflectron ion mode; 2,000 subspectra were integrated.

Moreover, the elastinolytic activity of TrCB2 was confirmed by digestion of soluble bovine elastin (Sigma E6527). The reaction was performed in 20 μl of CPB pH 6 containing 4 μg of TrCB2 and 40 μg of soluble elastin. Controls run in parallel either contained 50 μM E-64 or were free of the enzyme. Overnight incubation at 37 °C was followed by addition of non-reducing SDS–PAGE sample buffer, the samples were boiled for 3 min. and electrophoresed in an 8–16% linear gradient precast gel (Bio-Rad).

## 3 Results

### 3.1 Sequence analysis of the full-length TrCB2 cDNA clone and predicted protein

**Fig. 1:**
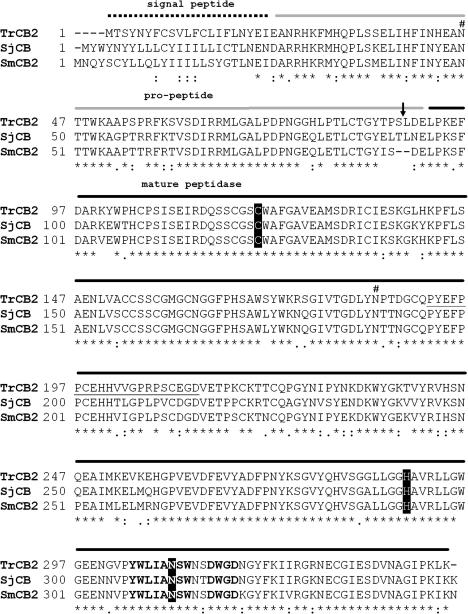
Multiple alignment of cathepsin B2 from *Trichobilharzia regenti* (TrCB2; GenBank Accession No. EF682129) with cathepsin B2 from *Schistosoma japonicum* (SjCB; GenBank Accession No. AY226984) and *Schistosoma mansoni* (SmCB2; GenBank Accession No. AJ312106). Above the sequences, the dotted line refers to the signal peptide, grey line to the pro-peptide and black line to the predicted mature enzyme. Below the sequences, identities across all three sequences are indicated by asterisks, one or more conservative substitutions are indicated by colons, non-conservative changes are represented by points. The conserved His, Cys and Asn residues forming the catalytic active site are shaded in black. Space symbol indicates the possible *N*-glycosylation site and arrow depicts the cleavage site between the pro-domain and mature peptidase as experimentally determined by N-terminal Edman degradation of expressed recombinant TrCB2. The ‘occluding loop’ unique to cathepsin B enzymes is underlined. The slightly modified ‘haemoglobinase motif’, ascribed to those cathepsins B which are able to cleave haemoglobin, is indicated in bold face.

The open reading frame (ORF) of TrCB2 is made up of 1,032 bp and encodes a pre-pro-enzyme of 344 amino acid residues with a calculated mass of 38.5 kDa. The predicted polypeptide has significant sequence similarity with cathepsins B2 from human schistosomes (78% to *S*. *japonicum* and 77% to *S*. *mansoni*) as shown in Fig. 1. Among the 50 bacterial clones screened, no sequence coding for another isoform of the enzyme was found. A potential signal sequence cleavage site was detected between amino acids at positions 21 and 22, according to SignalP v3.0 software. This would result in a pro-enzyme 323 amino acid residues long with a calculated mol. wt. of 36 kDa and an estimated pI of 6.5. The predicted mature enzyme has 253 amino acid residues, a calculated mol. wt. 28.5 kDa, p*I* 5.9 and starts with an amino acid at position 92. Nevertheless, the results from Edman degradation showed that the recombinant peptidase was cleaved into the mature form three amino acids upstream from the predicted site, between Ser^88^ and Leu^89^. Two potential *N*-glycosylation sites (Asn-Xaa-Thr/Ser) were identified at amino acid positions 46 and 185 of the pre-pro-enzyme. The conserved residues Cys^32^, His^201^ and Asn^221^ forming the typical catalytic triad of the family C1 of Clan CA cysteine peptidases (Turk et al., 2000) are present (shaded in black in Fig. 1). Also, the sequence contains a unique motif (YWLIANSWxxDWGE) ascribed to those cathepsins B thought to serve as haemoglobinases in blood-feeding helminths (Baig et al., 2002), except that the terminal glutamic acid (E) has been replaced by aspartic acid (D) in TrCB2 sequence. The nucleotide sequence for *T. regenti* cathepsin B2 has been deposited in the GenBank database (GenBank Accession No. EF682129). The amino acid sequence of this protein can be accessed through NCBI Protein Database (NCBI Accession No. ABS57370). The NC-IUBMB enzyme classification of TrCB2 is EC 3.4.22.1.

### 3.2 Functional expression and purification of recombinant TrCB2

**Fig. 2:**
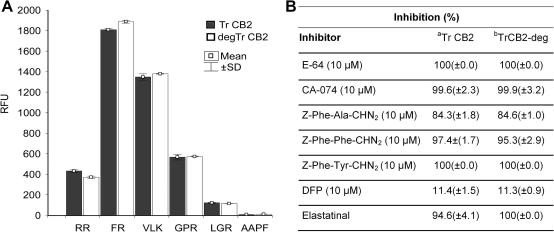
Fluorogenic peptide substrate hydrolysis and inhibition studies with de- and glycosylated forms of recombinant TrCB2 at pH optimum 6.0. (A) Peptidolytic activity compared with a spectrum of aminomethylcoumarine-labelled substrates. (B) Effect of cysteine and serine peptidase differentiating inhibitors on peptidolytic activity of TrCB2 with the cathepsin B-specific substrate RR. Composition of substrates is presented in single-letter aminoacid code. Values are means of three independent triplicate assays ± SD. RFU, relative fluorescent unit.

**Fig. 3:**
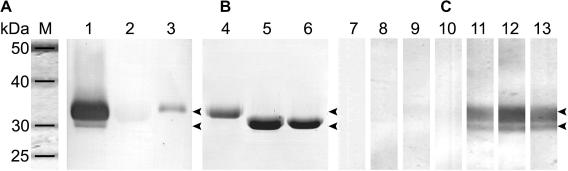
Detection of purified recombinant TrCB2 by SDS–PAGE, ligand and Western blotting. (A) Labelling of TrCB2 by the cysteine peptidase-specific probe DCG-04 on blot. Lane 1, DCG-04 binding to the active site of the enzyme; lane 2, binding of DCG-04 inhibited by cysteine peptidase inhibitor E-64; lane 3, weak reaction of DCG-04 with heat-inactivated enzyme. (B) Shift in the molecular size in SDS–PAGE of yeast-produced TrCB2 after deglycosylation with endoglycosidase F1. Lane 4, non-treated enzyme; lane 5, endoglycosidase F1 treated enzyme; lane 6, deglycosylation performed in the presence of E-64. (C) Reaction of mouse and rabbit sera raised against TrCB2 and SmCB2, respectively, on blots of recombinant glycosylated TrCB2. Lanes 7 and 8, controls with buffer instead of primary antibodies; lanes 9 and 10, pre-immune mouse and rabbit sera; lane 11, serum against glycosylated TrCB2; lane 12, serum against deglycosylated TrCB2; lane 13, serum against SmCB2. Note the strong reaction of DCG-04 and immune sera with the major 32 kDa glycosylated band of yeast-produced TrCB2; reaction also occurs with a minor 30 kDa band of supposedly non-glycosylated TrCB2 produced by yeast, which is not visible in Coomassie stained gel (lane 4). TrCB2 and SmCB2, cathepsins B2 of *Trichobilharzia regenti* and *Schistosoma mansoni*, respectively.

TrCB2 was expressed in the methylotropic yeast *P*. *pastoris* as a pro-enzyme, however, it had already undergone self-processing to a mature active peptidase in yeast medium as confirmed by the results of N-terminal sequencing (Fig. 1). The activity of the enzyme was confirmed by fluorometric and inhibition assays (Fig. 2). In addition, the cysteine peptidase-specific probe DCG-04 bound specifically to a 32/30 kDa doublet on ligand blots (Fig. 3) and mass spectrometry of the SDS–PAGE-resolved proteins confirmed the identity of the expression product to be cathepsin B (not shown).

The enzyme was purified from the medium in a single step by cation-exchange FPLC. The column fraction containing the greatest hydrolytic activity against cathepsin B-specific substrate Z-Arg-Arg-AMC was subjected to SDS–PAGE. Although the expected molecular mass of mature TrCB2 was 28.5 kDa, a band migrating at 32 kDa appeared in the gel. The glycosylation state of the secreted recombinant mature enzyme was examined by treatment with endoglycosidase F1, resulting in a significant decrease in molecular mass to 30 kDa in SDS–PAGE (Fig. 3).

### 3.3 pH optimum, peptidase assays, inhibition studies and S_2_ subsite specificity

For both glycosylated and deglycosylated forms of TrCB2, the pH optima for hydrolysis of Z-Arg-Arg-AMC were 6.0. At pH values below 4.0 and above 6.5 only slight hydrolysis was detected for both forms of TrCB2 (not shown).

As anticipated, Z-Arg-Arg-AMC, Z-Phe-Arg-AMC and Boc-Val-Leu-Lys-AMC were degraded, whereas activity with serine peptidase substrates Boc-Leu-Gly-Arg-AMC and Suc-Ala-Ala-Pro-Phe-AMC was only negligible. Z-Gly-Pro-Arg-AMC, usually used for trypsin-like peptidase activity detection, was hydrolysed by TrCB2 at a similar level as Z-Arg-Arg-AMC dipeptide. No significant differences in activity were observed between de- and glycosylated TrCB2 with particular substrates (Fig. 2). All cysteine peptidase-selective inhibitors used had remarkable inhibitory effects on the activity of TrCB2 with Z-Arg-Arg-AMC substrate. Among serine peptidase inhibitors, only elastatinal appeared to be potent (Fig. 2).

**Fig. 4:**
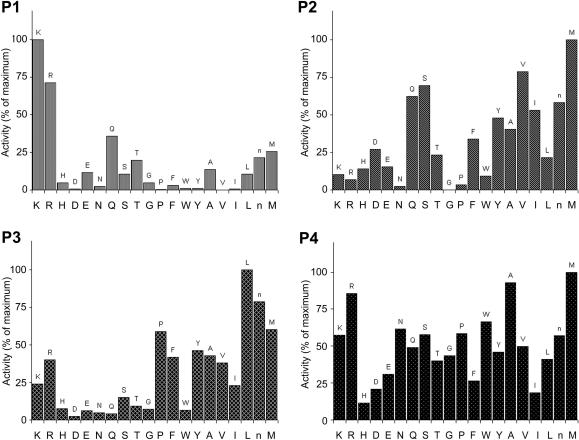
Preference profiles of recombinant TrCB2 using a complete diverse positional scanning synthetic combinatorial peptide substrate library. S_2_ specificities of the enzyme to corresponding P1–P4 peptide amino acid positions were determined with a library in which randomised positions were incorporated by addition of the isokinetic mixture of 20 amino acids. The amino acids are designated by the single-letter code on the *x*-axis (n, norleucine). The *y*-axis is a 100% scale of picomolar fluorophore produced per second.

The positional scanning of the synthetic combinatorial library at optimal pH employed to probe the S_2_ specificities clearly showed that recombinant TrCB2 displays partial preferences for Lys or Arg at position P_1_, Met > Val > Ser > Gln at P_2_ and Leu and other hydrophobic amino acids at P_3_ of peptide substrates. No obvious preference for an amino acid residue was noted at P_4_ (Fig. 4).

### 3.4 Immunoreactivity of TrCB2

Both de- and glycosylated forms of recombinant TrCB2 gave rise to four mouse polyclonal antisera. Three of those reacted positively with a protein doublet of 32 and 30 kDa on immunoblots of recombinant TrCB2. In addition, cross-reactivity of recombinant TrCB2 with rabbit monospecific polyclonal antiserum against cathepsin B2 from *S. mansoni* (anti-SmCB2) was detected (Fig. 2).

**Fig. 5:**
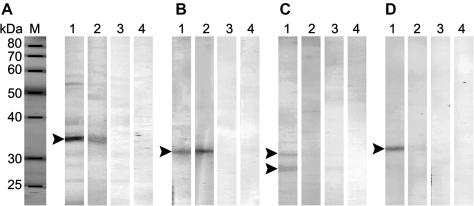
Reaction of mouse antiserum against TrCB2 and rabbit antiserum against SmCB2 with extracts from various stages of *Trichobilharzia regenti* and *Schistosoma mansoni* life cycles. (A) Cercarial extract of *T. regenti*, (B) cercarial extract of *S. mansoni*, (C) schistosomular extract of *T. regenti* and (D) extract from adult *S. mansoni* were resolved by reducing SDS–PAGE, electroblotted to polyvinylidene difluoride membrane and incubated with serum from a mouse immunised with TrCB2 (lanes 1), a rabbit immunised with SmCB2 (lanes 2), a non-immunised mouse (lanes 3) and from a non-immunised rabbit (lanes 4). Arrowheads show presumptive cathepsin B2 bands.

The anti-TrCB2 sera specifically recognised putative cathepsin B2 of ∼34 kDa on Western blots of *T. regenti* cercariae (Fig. 5A, lane 1) and two proteins of schistosomula at 31 and 27 kDa (Fig. 5C, lane 1). Anti-TrCB2 also reacted with an ∼31 kDa protein from cercariae and an ∼31 kDa band from adults of *S. mansoni* (Fig. 5B and D, lanes 1). Reciprocally, rabbit anti-SmCB2 specifically recognised particular proteins in extracts of both parasite species (Fig. 5A–D, lanes 2).

**Fig. 6:**
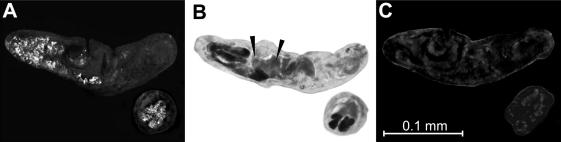
Immunolocalisation of cathepsin B2 in longitudinal and transverse sections of cercariae of *Trichobilharziaregenti* by indirect immuno-fluorescence microscopy. (A) Sections incubated with mouse anti-TrCB2 and then with AlexaFluor-labelled anti-mouse IgG. Positive reaction is apparent in post-acetabular penetration glands and their ducts. (B) Section of *T. regenti* cercariae was stained with H & E solution. Post-acetabular glands and their ducts appear in black. Arrowheads point to the circumacetabular glands. (C) Control with pre-immune mouse serum; no reaction is visible.

By means of immuno-fluorescence confocal microscopy with specific anti-TrCB2 serum, the enzyme was localised in post-acetabular penetration glands of *T*. *regenti* cercariae (Fig. 6). No signal was observed in other parts of cercarial bodies.

### 3.5 Processing of macromolecular natural substrates by recombinant TrCB2

**Fig. 7:**
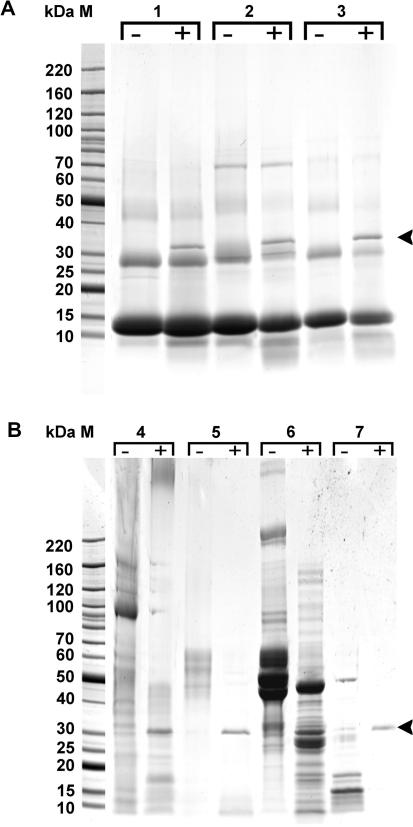
Digestion of selected natural substrates by recombinant cathepsin B2 from *Trichobilharzia regenti* (TrCB2). The substrates at a final concentration of 0.2 mg/ml were incubated in the presence (+) or absence (−) of recombinant TrCB2 (1 μg) in 50 μl of citrate–phosphate buffer pH 6 containing 2 mM DTT at 37 °C overnight. Control reactions (−) contained CPB instead of the enzyme. Aliquots (20 μl) of the resulting hydrolysates were separated by electrophoresis and stained with Coomassie brilliant blue. Substrates used: Gel (A) haemoglobin, lanes 1, from duck; lanes 2, bovine; lanes 3, turkey; Gel (B) lanes 4, collagen type IV; lanes 5, keratin; lanes 6, fibrinogen type III; lanes 7, myelin basic protein. Arrowheads indicate bands corresponding to recombinant TrCB2. Note that all substrates were efficiently degraded, except for haemoglobins hydrolysed only at a negligible rate.

**Fig. 8:**
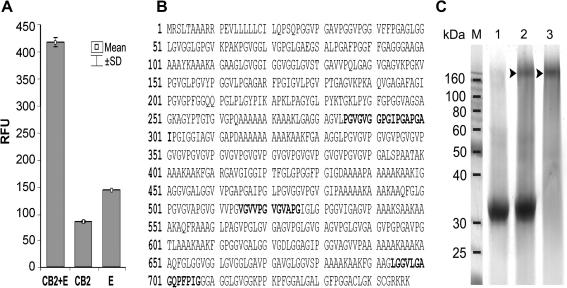
Digestion of elastin by recombinant cathepsin B2 from *Trichobilharzia regenti* (TrCB2). Insoluble (A and B) or soluble (C) elastin in citrate-phosphate buffer pH 6 was incubated with recombinant TrCB2. (A) The reaction was stopped by adition of trichloracetic acid, centrifuged and an aliquot combined with a 0.2 M sodium borate buffer, pH 8.5 and fluorescamine solution. The fluorescence of labelled peptides was measured at *λ*_ex_ = 390 nm and *λ*_em_ = 480 nm. Graph shows the difference in fluorescence of sample containing both substrate and enzyme compared with controls with either the enzyme or substrate alone, indicating the presence of new peptides originating from elastin digested by TrCB2. Values are means of triplicate assays ± SD. E, elastin; RFU, relative fluorescence unit. (B) The samples were concentrated and applied directly on nano-reverse phase-HPLC-MALDI-TOF-TOF. Bolded amino acids show partial sequences deciphered by mass spectrometry analysis as unique for sample containing both elastin and TrCB2. (C) SDS–PAGE proof of soluble elastin degradation by the 32 kDa TrCB2 (lane 1); no hydrolysis was observed in the presence of E-64 inhibitor of cysteine peptidases (lane 2); soluble elastin band (lane 3) is indicated by an arrowhead.

Most of the selected natural protein substrates used in the study (skin proteins keratin, collagen and elastin, neural myelin basic protein, serum fibrinogen) were efficiently degraded by recombinant TrCB2 at pH 6.0. Haemoglobinolytic activity of the enzyme tested with turkey, bovine and duck haemoglobins was negligible (Figs. 7 and 8).

## 4 Discussion

Following our previous biochemical studies, here we employed a molecular approach to gain a more extensive insight into the proteolytic equipment of bird schistosome cercariae. A novel cathepsin B peptidase from the nasal neurotropic fluke *T. regenti* was identified, functionally expressed and characterised; according to its high sequence similarity to cathepsins B2 of human schistosomes, the enzyme was termed TrCB2. Sequencing of the ORF of TrCB2 revealed that all of the characteristic features of the C1 family of Clan A cysteine peptidases were present, most notably the catalytic triad (Cys^32^, His^201^ and Asn^221^) and an occluding loop (residues Pro^104^–Cys^130^), the latter presumably providing the enzyme with peptidyl-dipeptidase (exopeptidase) activity (Musil et al., 1991). The recombinant *T. regenti* cathepsin B2 was obtained in an active mature form, implying self-activation by slitting-off the pro-domain under the conditions of yeast media and obviating the need for protein refolding and/or activation steps. Accordingly, the straightforward expression and purification of mature cathepsin B2 from *S. mansoni*, which is the most related peptidase to TrCB2 regarding sequence similarity, was reported (Caffrey et al., 2002).

The expected size of mature TrCB2 was 28.5 kDa. However, recombinant TrCB2 isolated from *P. pastoris* culture media was determined to have a greater molecular size of 32 kDa by SDS–PAGE. The difference between predicted mass and the mass of migrating expression product could be explained partially by the cleavage of the pro-enzyme three amino acids upstream of the predicted site. Alternatively, the difference could be the result of yeast-produced Asn-linked *N*-glycosylation (Bretthauer and Castellino, 1999). Indeed, after incubation of the 32 kDa TrCB2 with endoglycosidase F1 cleaving oligomannoside and hybrid oligosaccharides, the molecular mass of the deglycosylated protein significantly decreased from 32 to 30 kDa. More sensitive ligand and Western blotting analyses showed a double band of 32 and 30 kDa prior to deglycosylation, the latter being minor. This suggests that a minority of TrCB2 molecules produced by yeast can be non-glycosylated. Although hyperglycosylation by yeast may have resulted in an inactive recombinant protein, both de- and glycosylated forms of TrCB2 were enzymatically active, not differing significantly in their specific activities and optimum pH 6.

Besides cathepsin B-specific substrate Z-Arg-Arg-AMC, TrCB2 was able to hydrolyse other cysteine peptidase-specific substrates: Z-Phe-Arg-AMC and Boc-Val-Leu-Lys-AMC. Z-Gly-Pro-Arg-AMC, commonly used for serine peptidase detection, was also sensitive to hydrolysis; this could be explained by the presence of highly preferred Arg at P_1_ position of the substrate. The preferences of TrCB2 for particular amino acids in positions P_1_ through P_4_ (P_1_: Lys > Arg; P_2_: Met > Val > Ser > Gln; P_3_: Leu and other hydrophobic aminoacids; P_4_: X) conform to those mapped for *S. mansoni* cathepsin B2 using similar libraries (Choe et al., 2006). Cathepsin B-like peptidases of parasites were confirmed by the complete diverse PS-SCL assays to have broad and overall substrate specificity similar to other cysteine cathepsins. A high substrate promiscuity of cathepsins B has been suggested (Spiess et al., 1994; Khersonsky et al., 2006).

The peptidolytic activity of TrCB2 was efficiently inhibited by the general Clan CA cysteine peptidase inhibitor E-64, as well as by inhibitors specific for cathepsins B and L (Z-Phe-Ala-CHN_2_ and Z-Phe-Phe-CHN_2_) and cathepsin B (CA-074). Z-Phe-Tyr-CHN_2_ also fully inhibited TrCB2 at the given concentration, although it is often mentioned as being specific for cathepsins L rather than cathepsins B (Klose et al., 2006). Strikingly, elastatinal, a specific inhibitor of neutrophil and pancreatic elastases (serine class peptidases), showed a level of inhibition comparable to inhibitors specific for cysteine peptidases. This phenomenon was reported by Kašný et al. (2007), who suggested that, considering the structure of elastatinal [*N*-(Na-Carbonyl-Cpd-Gln-Ala-al)-Leu] and the structure of the cysteine peptidase inhibitor Z-Phe-Ala-CHN_2_ (which was also a good inhibitor of TrCB2 activity in this study), the aldehyde located on Ala_2_ situated in P_1_ position of elastatinal is probably able to inhibit cysteine peptidase activity. The activity and inhibition data presented here corroborate previously reported results on cathepsins B of schistosome flukes (Caffrey et al., 2002; Dvořák et al., 2005).

The immunocross-reactivity of proteins among closely related schistosome species is quite common, due to the similarity of shared epitopes recognised by the host immune system. Consequently, the detected cross-reactivity of anti-SmCB2 with recombinant TrCB2 was not surprising because the amino acid sequence similarity of TrCB2 and SmCB2 was as high as 77%. The conspicuous differences between molecular sizes of the cross-reacting proteins recognised by the anti-TrCB2 and anti-SmCB2 sera in the extracts from different life stages of *T. regenti* and *S. mansoni* could be explained by the various kinds or extent of post-translational modifications, most probably glycosylation. Although cathepsin B2 has been reported only from adults and schistosomula of *S. mansoni*, in our study, the presence of SmCB2 was identified for the first time, to our knowledge, in *S. mansoni* cercariae via the reaction of an ∼31 kDa band on Western blot of cercarial extracts with sera raised against both SmCB2 or TrCB2. In *S*. *mansoni*, cathepsin B2 is found in tegumental tubercles and parenchyma of adult males and to a lesser extent in female worms. Therefore, it has been suggested to be likely lysosomal and act at the parasite–host interface by participating in the turnover of tegumental proteins, the degradation of endocytosed proteins and/or operating in a protective capacity (Caffrey et al., 2002). However, no studies have been performed on the capability of SmCB2 to cleave relevant protein substrates. In the case of *T*. *regenti*, immunohistochemistry of cercariae using anti-TrCB2 antibodies localised the enzyme to the post-acetabular penetration glands. These are specialised cells, whose secretions help penetrating larvae to disrupt host surface epithelia and underlying tissues of the skin. Our finding outlines a probable role of this enzyme in aiding cercarial penetration of their hosts.

In vitro studies showed that recombinant TrCB2 is capable of digesting skin/nervous tissue macromolecules (collagen, elastin, keratin and myelin basic protein), supporting the above-mentioned putative role of TrCB2 in host skin invasion and subsequent migration through nervous tissues. Although it has been widely assumed that skin penetration by schistosomes is mediated mainly by serine peptidases of elastase-like type localised in circumacetabular penetration glands (Salter et al., 2000, 2002), the participation of cathepsin B-like cysteine peptidases in this process has been previously hypothesised (Dalton et al., 1997) and is supported by our studies. The potency of cathepsins B to cleave elastin has been previously confirmed with human cathepsin B, although cathepsin L had a higher activity on this substrate (Mason et al., 1986). Here we believe we report the first demonstration of elastinolytic activity by a parasite cathepsin B.

In our experiment with insoluble elastin, DTT was used to protect the nucleophilic thiol group of cysteines in the cysteine peptidase-active site. We are aware that it has also a denaturating effect on some proteins. It serves as a reducing agent, disrupting disulphide bonds within polypeptide molecules or between protein subunits, thus breaking up tertiary or quaternary structures. However, in the case of elastin, disulphide bridges do not occur between particular tropoelastin molecules forming the elastin polymer. These are cross-linked via desmosine/isodesmosine bonds derived from specific lysines. Only one intramolecular disulphide bridge is present within the tropoelastin subunit; this interconnects the only two cysteines of the mature tropoelastin which are located near the C-terminus. The intrachain disulphide bond in the carboxy terminal domain of tropoelastin stabilises a loop structure and positively charged pocket (Brown et al., 1992). We think that the change in the tertiary structure of the C-terminus could hardly affect the accessibility of the rest of the elastin molecule for proteolytic cleavage by TrCB2 which occurred in glycine-, valine- and proline-rich areas of β-spirals of the elastin molecule (Rucker and Dubick, 1984) as detected by mass spectrometry of newly arisen peptides. Furthermore, the reducing power of DTT is limited to pH values above ∼7, since only the negatively charged thiolate form –S^−^ is reactive (the protonated thiol form –SH is not); the p*K*_a_ of thiol groups is typically ∼8.3 (Cleland, 1964). Our experiment was performed at pH 6. Finally, the cleavage of “soluble elastin” was performed in DTT-free buffer. Therefore, we believe that we can affirm that TrCB2 possesses true elastinolytic activity. The question remains, what is the rate of *T*. *regenti* cathepsin B2-specific activity in comparison to the elastase of *S*. *mansoni*.

In the case of cercariae of *T*. *regenti*, cercarial secretions display a high cathepsin B-like activity and moreover, the presence of an elastase orthologue was not confirmed using biochemical (Kašný et al., 2007), immunological (Mikeš et al., 2005) or molecular approaches (Dolečková et al., 2007). Thus, the competence of TrCB2 to cleave elastin (one of the major components of vertebrate skin) indicates that this enzyme could functionally substitute for the role of the chymotrypsin-like serine peptidase referred to foremost as “cercarial elastase” in *S. mansoni*.

The evidence presented above leads us to suggest that during skin invasion, cercariae of *T. regenti* use enzymatic tools which are distinct from those of *S. mansoni*. These rather resemble the situation in cercariae of *S*. *japonicum*, where no elastase-like serine peptidases have been identified and where, on the contrary, a high level of cathepsin B activity is detected. It has been proposed by these authors that cysteine peptidases represent an archetypal tool for tissue invasion among primitive metazoa and the use of serine peptidases arose later in schistosome evolution (Dvořák et al., 2008).

In order to determine possible substrates for peptidase activity released during schistosomula migration through nervous tissues, we observed that myelin basic protein is a far better substrate for hydrolysis by recombinant TrCB2 than haemoglobin. Although TrCB2 contains a sequence motif similar to that contained in cathepsin B involved in the digestion of haemoglobin by blood-feeding helminths (Baig et al., 2002), this motif is slightly changed by the substitution in the last amino acid, as in the case of SmCB2 and SjCB2 (see Fig. 1). These results imply that TrCB2 is not involved in in vivo degradation of haemoglobin but is employed against nervous system material (e.g. myelin basic protein) in order to facilitate parasite migration through the nerves.

To elucidate the roles of cathepsin B-like cysteine peptidases throughout life cycles of different schistosome species, their expression in particular parasite stages and tissues should be proved and detailed functional experiments need to be conducted.
